# Bone Metastasis Pain, from the Bench to the Bedside

**DOI:** 10.3390/ijms20020280

**Published:** 2019-01-11

**Authors:** Federica Aielli, Marco Ponzetti, Nadia Rucci

**Affiliations:** Department of Biotechnological and Applied Clinical Sciences, University of L’Aquila, 67100 L’Aquila, Italy; federica.aielli@univaq.it (F.A.); mp.univaq@gmail.com (M.P.)

**Keywords:** bone pain, osteoclasts, osteoblasts, bone metastasis, skeletal-related events

## Abstract

Bone is the most frequent site of metastasis of the most common cancers in men and women. Bone metastasis incidence has been steadily increasing over the years, mainly because of higher life expectancy in oncologic patients. Although bone metastases are sometimes asymptomatic, their consequences are most often devastating, impairing both life quality and expectancy, due to the occurrence of the skeletal-related events, including bone fractures, hypercalcemia and spinal cord compression. Up to 75% of patients endure crippling cancer-induced bone pain (CIBP), against which we have very few weapons. This review’s purpose is to discuss the molecular and cellular mechanisms that lead to CIBP, including how cancer cells convert the bone “virtuous cycle” into a cancer-fuelling “vicious cycle”, and how this leads to the release of molecular mediators of pain, including protons, neurotrophins, interleukins, chemokines and ATP. Preclinical tests and assays to evaluate CIBP, including the incapacitance tester (in vivo), and neuron/glial activation in the dorsal root ganglia/spinal cord (ex vivo) will also be presented. Furthermore, current therapeutic options for CIBP are quite limited and nonspecific and they will also be discussed, along with up-and-coming options that may render CIBP easier to treat and let patients forget they are patients.

## 1. The Healthy Bone Tissue

Despite its hard nature, due to the deposition of hydroxyapatite crystal in the extracellular matrix, bone undergoes several cycles of renewal during the life of each individual, which guarantee proper mechanical performance as well as bone regeneration during fractures repair. This amazing and quite exclusive property, called bone remodelling, can be accomplished by means of three resident cell types: osteocytes, osteoblasts and osteoclasts [1].

Osteocytes are the most abundant cell type in the bone, comprising more than 90% of bone cells [2]. They come from the osteoblast lineage, and they are essentially osteoblasts that have surrounded themselves in bone matrix. Osteocyte’s most recognised role is that of mechanosensors, but many other functions are emerging [3]. As an example, osteocytes can depose and resorb bone around them in particular conditions, such as hypercalcemia and lactation, respectively [3,4]. Also, osteocytes have endocrine functions and can regulate renal phosphate wasting, as well as the metabolism of the two other cell types in the bone [2,3,5].

Osteoblasts originate from bone marrow mesenchymal stem cells (BMMSCs), after their commitment towards an osteo/chondro progenitor. A crucial role in osteoblast differentiation is recognised to the Wingless-type MMTV integration site family (WNT) signalling pathway [6,7,8]. In particular, the binding of Wnt proteins (the family includes at least 19 different isoforms) to frizzled (Fzd) receptor and to Lrp5/Lrp6 coreceptors [9] promotes the stabilization and accumulation of the downstream protein β-catenin, which can translocate into the nucleus and activate the lymphoid-enhancing factor/T cell factor (Lef/Tcf) transcription factor, thus switching on the transcription of key genes for osteoblast differentiation. Among them, Runt-related transcription factor 2 (Runx2) has a prominent role [10,11]. This transcription factor is a direct target of Wnt/β-catenin pathway and, in turn, promotes the expression of the osteoblast marker alkaline phosphatase (ALP) and of the bone matrix proteins collagen I, bone sialoprotein (BSP) and osteocalcin (OCN). Another molecule that may be important in the commitment towards osteo- rather than chondro-progenitors is CD146: its expression has been shown to be able to dissuade BMMSCs from differentiating into osteoprogenitors [12]. The bone marrow microenvironment is of course crucial for BMMSCs commitment. However, newly-developed scaffolds derived from muscle seem to be able to direct differentiation and commitment of these stem cells, which might be important for regenerative medicine in the future [13].

The main role for osteoblasts is to produce bone matrix by secreting collagen and noncollagenous proteins, such as osteopontin (OPN), osteonectin, BSP, dentin matrix protein (DMP)-1 and OCN. The newly formed bone matrix, called osteoid, is subsequently mineralised by the precipitation of hydroxyapatite crystals, also this process being orchestrated by osteoblasts [14]. Importantly, cancerous transformation of osteoblasts gives rise to osteosarcoma [15], while osteosclerotic bone metastases derive from osteoblasts reprogramming [16].

Another crucial role for osteoblast is to regulate osteoclasts differentiation. Osteoclasts are giant multinucleated cells belonging to the monocyte-macrophage lineage, which resorb bone [17]. Two transcription factors—PU-1 and microphthalmia transcription factor (MITF)—are crucial to fuel the commitment of haematopoietic stem cells towards a common macrophage/osteoclast progenitor, which expresses the receptor for macrophage-colony stimulating factor (M-CSF), c-fms [18,19]. Osteoblasts produce M-CSF, which has a promitotic and survival effect on osteoclast precursors [20] and, most importantly, increases the expression of the RANK receptor on osteoclast precursors [21]. This is a crucial stage for osteoclast differentiation, which allows the activation of the RANKL/RANK signalling cascade [22]. RANKL belongs to the TNF superfamily [23] and is produced by osteoblasts, osteocyte and stromal cells mainly as a transmembrane form and to a lesser extent released as a soluble form after enzymatic cleavage. It binds to RANK receptor, expressed by osteoclast precursors, thus stimulating their fusion and differentiation towards mature osteoclasts by a mechanism nuclear factor ĸ-light-chain-enhancer of activated B cells (NF-ĸB) mediated [22,24]. NF-kB stimulates the expression of another important transcription factor, the nuclear factor of activated T cells, cytoplasmic, calcineurin-dependent (NFATc1) [25], while RANKL also activates tumour necrosis factor receptor-associated factor (TRAF)-6 leading to c-Src engagement, which in turn mediates osteoclastogenesis through the activation of phosphoinositide 3-Kinase (PI3K)/ protein kinase B (Akt) pathway [26].

Of note, inflammatory cytokines, such as tumour necrosis factor (TNF)-α, Interleukin (IL)-1β and IL-6 can strongly induce osteoclastogenesis by activating the NF-kB pathway [27,28,29].

### The Virtuous Cycle in the Physiology of Bone

Proper bone mass is the result of a correct balance between bone degradation, accomplished by osteoclasts, and the subsequent deposition of new bone matrix by osteoblasts, thus accomplishing the bone remodelling process, known as the “virtuous circle”. The first stage requires the activation, by means of different stimuli (i.e., systemic/paracrine factors, mechanical loading changes and microfractures) of the lining cells, which usually cover the bone surface under resting conditions. These cells in turn secrete factors attracting osteoclast precursors in the site that need to be resorbed, stimulating osteoclast differentiation [30]. Once activated, the osteoclasts degrade the bone matrix, with a consequent release of calcium (Ca^2+^) ions as well as of factors usually stored in the bone, which are chemoattractant for osteoblasts in the reabsorbed area [31]. This is the fastest phase of the bone remodelling process, ending with osteoclast apoptosis. At this point, macrophage-like cells, called reverse cells, incorporate the debris produced during bone degradation and contribute to the recruitment of osteoblasts, which refill the resorbed lacunae with new bone.

In normal conditions all these phases are strictly regulated by hormones as well as by a tight cross-regulation between osteoclasts and osteoblasts. As will be stated in the next paragraphs, any deregulation of this “virtuous circle” is the leading cause of many bone-related pathologies, including bone metastases, in which the conversion of the virtuous cycle in a “vicious” one represents a point of no return [32].

## 2. Molecular Mechanisms of Bone Metastases

Bone metastasis prevalence is increasing more and more over the years. This is likely a consequence of an improvement of life expectancy in oncologic patients [33]. Indeed, although bone still represents the preferential site of metastasis for breast and prostate cancers, with an incidence up to 70 and 90%, respectively, other cancers are prone to colonise this tissue, such as lung, kidney and thyroid [33,34]. Usually, the axial skeleton, especially the vertebral column, is preferred by cancer cells as a site of metastases. The consequences are devastating, impairing both life quality and expectancy, due to the occurrence of the skeletal-related events (SREs). These are characterised by bone fractures, hypercalcemia, frequently the leading cause of death, spinal cord compression due to infiltrating vertebral metastases and severe bone pain [35]. Of note, several clinical trials demonstrated that bone pain and use of analgesic significantly increase within the six months preceding the onset of SREs [36,37].

From a clinical point of view, bone metastasis can be classified as osteosclerotic or osteolytic, according to X-ray features [38]. The former appears as radiopaque spots, because of an exacerbated bone deposition by osteoblasts. However, this new bone is of poor quality because of the disorganised disposition of collagen I fibrils (“woven” bone). Almost all bone metastases induced by prostate cancers show osteosclerotic features. In contrast, breast cancer-induced bone metastases are preferentially osteolytic, as a result of exacerbated osteoclast-mediated bone erosion. There is however a third class, where osteolytic and osteosclerotic bone metastases coexist in the same district (i.e., mixed bone metastases) which can be induced by both breast and prostate cancers [38,39]. Regardless this classification, the development of bone metastases is always the result of a dramatic disruption of the bone remodelling process, fuelled by tumour cells colonisation of the bone/bone marrow once egresse from the bloodstream. Although it has been over 100 years, the “seed and soil theory” of Steven Paget still represents a milestone in the bone metastases field [40]. Like the seed needs proper soil to implant and grow, tumour cells need biochemically and physiologically favourable conditions in the host tissue to survive and colonise it.

### The “Vicious Cycle”

**Figure 1:**
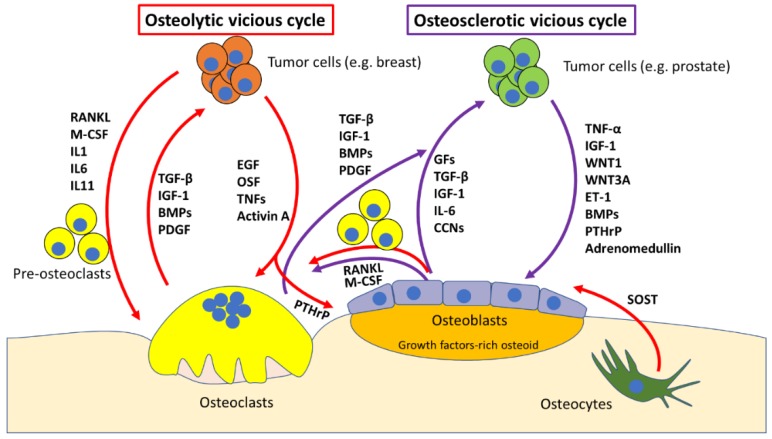
Cartoon representing the osteolytic and osteosclerotic vicious cycles. Osteolytic cycle: osteolysis-generating cancer cells (e.g., breast) migrate to the bone marrow and start secreting osteoclast-stimulating factors such as epithelial growth factor (EGF), osteoclast stimulating factor (OSF), tumour necrosis factors (TNFs) and Activin A. In parallel, cancer cells also secrete osteoclast-differentiating factors, such as receptor activator of nuclear factor kB ligand (RANKL), macrophage-colony stimulating factor (M-CSF) and Interleukin (IL)-1, 6 and 11, which promote differentiation of pre-osteoclasts into osteoclasts. The bone matrix is rich in growth factors (GFs), transforming growth factor (TGF)-β, insulin-like growth factor (IGF)-1, bone morphogenic proteins (BMPs) and platelet-derived growth factor (PDGF). These factors get released when osteoclasts resorb bone, and they promote tumour growth, which closes the osteolytic vicious cycle. Osteocytes also take a crucial part in the process, secreting sclerostin (SOST) in response to osteolytic cancers (especially multiple myeloma), which inhibits osteoblast activity and the Wnt- β-catenin pathway Osteosclerotic cycle: osteosclerosis-generating cancer cells (e.g., prostate) migrate to the bone marrow and start secreting osteoblast-stimulating factors such as TNF-α, IGF-1, Wingless-type MMTV integration site (WNT)1, WNT3A, Endothelin (ET)-1, BMPs, parathyroid hormone-related peptide (PTHrP) and adrenomedullin. This stimulates osteoblast differentiation and activity. On one hand this leads to osteoblast-mediated osteoclastogenesis by increasing osteoblastic expression of RANKL and M-CSF, which causes the release of growth factors as discussed for the osteolytic cycle. On the other hand, osteoblasts themselves release a plethora of factors including GFs, TGF-β, IGF-1, IL-6 and chemokines (CCNs), which stimulate tumour growth, closing the osteosclerotic (which most of the times has an osteolytic component as well) vicious cycle.

When tumour cells migrate to the bone, they establish a pathological communication with bone cells, coming between osteoblasts and osteoclasts and their “virtuous cycle”, and hijaching it into what is called “vicious cycle” [32,41]. Mirroring the two kind of bone metastasis (i.e., osteosclerotic versus osteolytic) two types of vicious cycles can be identified (Figure 1). The osteosclerotic vicious cycle is typical of prostate cancer cells [42] which migrate to the bone, and alter the normal physiology of osteoblasts and osteoclasts, which results in the deposition of growth-factors-rich osteoid, which is then mineralised into low mechanical competence, non-remodelled bone [43]. Although osteosclerotic lesions are considered a hallmark of prostate cancer bone metastases, and X-ray evidence of osteosclerotic lesion is widely used as diagnostic tell for these events, osteosclerotic lesions are either preceded or can coexist with osteolytic lesions, in the same site or even in distant sites within the same patient [44,45]. A common hypothesis is that prostate cancer bone metastases first need to activate osteoclastic bone resorption, to then increase bone formation, which supports the clinical observation that Zoledronic acid, a bisphosphonate drug, and Denosumab, an anti-RANKL monoclonal antibody, are efficacious in the management of SREs of prostate cancer bone metastases and associated pain [46,47]. This is further supported by the notion that parathyroid hormone-related peptide (PTHrP), a known inducer of osteoblast-mediated osteoclastogenesis, is highly expressed in bone metastases from prostate cancer [48,49]. This factor acts through autocrine and paracrine signalling to increase cancer cell and osteoblast survival as well as osteolysis formation, and it is therefore a strong candidate as an osteoblastic vicious cycle molecular starter [50].

In addition to PTHrP, numerous molecular players in the establishment of the osteoblastic vicious cycle have been identified. Prostate cancer cells secrete osteoblast-stimulating factors, including adrenomedullin, platelet-derived growth factor (PDGF), insulin-like growth factors (IGFs) and members of the WNT signalling pathway, such as WNT1 and WNT3A [51]. In return, osteoblasts produce growth-factors that promote growth and survival of prostate cancer cells thus fuelling the vicious cycle. Nevertheless, the microenvironment of prostate cancer bone metastasis is very complex, and many other players such as Endothelin (ET)-1, which can also fuel the cycle and is a candidate therapeutic target [52], and vascular endothelium growth factor (VEGF), which increases osteoblast activity and cancer neoangiogenesis, are emerging [53].

Osteolytic bone metastases are the most common consequence of many neoplasms, such as breast and renal cancer [54]. It is well established that in osteolytic metastases, bone resorption is caused by a hyperactivation of osteoclasts by tumour cells rather than a direct action of cancer cells [32]. This process usually involves the activation of the NFkB pathway in osteoclast precursors. Consistently, the most important inflammatory cytokines—TNF-α, IL-1β and IL-6—can all be used by cancer cells to induce osteoclastogenesis by activating the NFkB pathway. However, the pathway that is most often regulated by osteolysis-generating cancer cells is the RANKL/osteoprotegerin (OPG)-RANK axis. This can happen directly, through cancer cells expression of RANKL (most common) or by exploiting osteoblasts and/or osteocytes, which are stimulated to produce RANKL through cancer-cell-released PTHrP [55] and in the case of osteocytes, to resorb bone directly [4,56]. As described above, activating osteoclast-mediated osteolysis allows the release of growth factors and chemokines stored into the bone matrix, such as TGF-β1, IGF-1, BMPs and FGFs, which fuel tumour growth and at the same time makes physical space for cancer cells.

Another important factor in bone marrow metastases is oxidative stress: for example, the known chemotherapeutic agent doxorubicin, although helpful as cancer drug, has been shown to cause bone loss due to induction of oxidative stress in osteoclasts in vitro, which causes an increase in their activity. TGF-β also seems to be involved in this process [57]. Consistently, treating neuroblastoma cells in vitro with an antioxidant can impair their growth [58].

Importantly, both the osteosclerotic and the osteolytic vicious cycles result in the release of many molecular mediators of pain, as described in the following paragraphs.

## 3. Molecular Determinants of Cancer-Induced Bone Pain (CIBP)

Cancer-related pain is arguably the most common consequences of this disease [59], significantly reducing quality of life and affecting the ability to complete everyday tasks and live a normal life. Among these, cancer-induced bone pain (CIBP) is one of the most prevalent, presenting as movement-related, constant or most commonly, in combination [60]. Bone metastases can then induce CIBP in several ways, many of which are still under investigation [61,62]. Indeed, bone is a richly innervated tissue, and sensitive neurons can be found in both the periosteum [63,64] and the bone marrow [65,66]. Among these, the most abundant are nociceptors—which mediate acute and chronic bone pain—which will be the focus of the present review article. At the molecular level, many key players have been identified, including acidity, nerve growth factor (NGF) and other neurotrophins, ILs and other inflammatory mediators, as well as up-and-coming factors that emerged in recent years.

Historically, the periosteum has been the focus of attention for most studies on bone nociception [63,64] probably due to its ease of access and stimulation. These studies revealed the generation of action potentials following sufficient mechanical stimulation of the periosteum. Studying the conduction velocities and the properties of these afferent neurons led to the finding that an overwhelming majority of the neural terminations innervating the periosteum were myelinated Aδ- and non-myelinated C-type. These two types of terminations are associated with breakthrough, quick onset pain or constant, burning, chronic pain, respectively. These neurons respond to pressure, and some of them are also sensitive to heat/cold shock and chemical stimulation with known algesic substances, such as bradykinin and potassium chloride [67]. Indeed, human subjects that were stimulated in the periosteal region of the appendicular skeleton reported pain as well [68].

Bone marrow pain has been less intensively investigated. What we know today about innervation of the bone marrow is that it is similar in composition to the periosteum. Most sensory terminals are consistent with conduction velocities of myelinated Aδ- and non-myelinated C-type terminals. However, at variance with the periosteum, no Aβ innocuous mechanoceptors and Golgi-Mazzoni corpuscles have been identified in the bone marrow to date. The classical activation signal of bone marrow nociceptors is an increase of intramedullary pressure. This was originally tested on dogs, by pressurising appendicular bone marrow using saline injections. The dogs assumed behaviour consistent with pain [69]. This is reflected by some human pathologies, including intraosseous engorgement syndromes [70,71] and bone metastases [72]. Consistent with the notion, relieving intraosseous pressure via fenestrations is able to significantly reduce bone marrow pain. This kind of pain is undoubtedly the most important one in the management of bone metastases, since most tumours are prone to metastasise to trabecular bone, which is always found in the medullar cavity.

**Figure 2:**
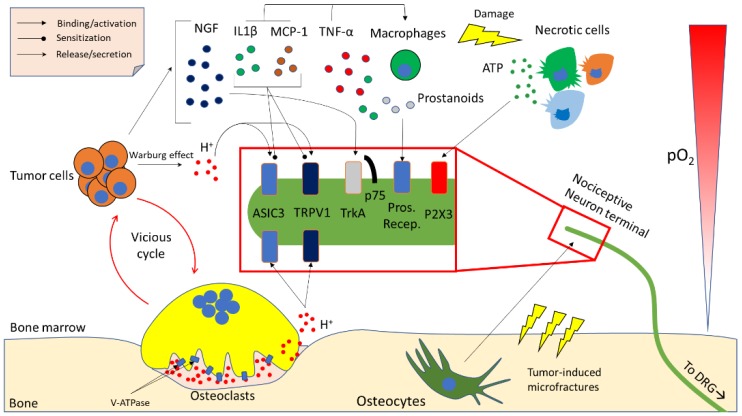
Cartoon representing the main cellular and molecular players in cancer-induced bone pain (CIBP). Partial pressure of O_2_ (pO_2_) decreases to approximately 50 mmHg moving from bone marrow sinusoids towards bone. When tumour cells are present in the bone marrow microenvironment, the low pO_2_, along with other factors, causes the switch from a mostly oxidative to a mostly glycolytic glucose metabolism, which results in the production of lactate and protons (H^+^) that are released in the microenvironment to keep intracellular pH stable (Warburg effect). Cancer cells also promote osteoclast formation and activity. Osteoclasts release protons into the resorption lacuna through the vacuolar-ATPase (V-ATPase). Protons may then leak towards the bone marrow microenvironment due to improper seal or apoptosis of the osteoclast. Protons are subsequently able to activate acid sensing ion channels (ASIC)-3, and transient receptor potential channel-vanilloid subfamily members (TRPV)-1, on nociceptive neuron terminals arising from dorsal root ganglia (DRGs), which causes the activation of pain pathways. Osteocytes can activate nociceptive neurons in different ways in response to tumour-induced microfractures, but they are mainly thought to release protons as signalling molecule, although other molecules such as neurotrophins have been proposed as osteocyte-derived pain-mediating factors. Tumour cells also secrete many factors that are able to activate or sensitise pain-mediating receptors, such as nerve growth factor (NGF), which binds the tropomyosin-related kinase (Trk)A-p75 receptor complex. Also, tumour-derived interleukin (IL)-1β, along with macrophage chemoattractant protein (MCP)-1 can sensitise ASIC3 and TRPV1 and activate and chemoattract macrophages. These latter cells further secrete IL-1β, MCP-1 and tumour necrosis factor (TNF)-α, which act as autocrine factors as well as induce cell death in the tissue. Macrophages also produce prostanoids, which activate nociception through prostanoid receptors. Because of the strong immune response, reactive oxygen species (ROS) generation, cancer-induced cytotoxicity and many other factors, cells in the bone marrow microenvironment undergo necrosis, which leads to the release of ATP. ATP can then act as a pain mediator by activating the purinergic P2X3 receptor on nociceptive neuron terminal.

Importantly, both periosteal and bone marrow nociceptors are often multimodal (i.e., can respond to different stimuli) and can start signalling in response to molecular and chemical mediators of pain, heat, cold, reactive oxygen species and acidity [63]. Much work has been done over the past years to characterise the mechanism of action of these pain mediators. A review of the most prominent findings is presented in the following paragraphs, and in Figure 2.

### 3.1. Acidity

Osteoclasts, and in particular conditions also osteocytes, use acidity to solubilise the mineralised fraction of the bone matrix, allowing ionic calcium and phosphates to be released into the bloodstream [73]. The acidification process must be tightly controlled to avoid protons leaking towards the bone marrow and having ineffective bone resorption. Physiologically, osteoclasts create a tightly sealed area using podosomes, limiting proton efflux to a discreet area between the osteoclast itself and the bone matrix, called the resorption lacuna [30]. When local bone resorption is complete, osteoclasts release the seal and move to a close area or undergo apoptosis. In normal conditions this process involves little or no proton leakage, and the relatively small number of osteoclasts guarantees that the pH of the bone marrow is not altered by bone remodelling. Cancer-induced osteoclastogenesis creates an acidity excess in the bone marrow by increasing absolute osteoclast number. Additionally, tumour cells also contribute to the acidification of the microenvironment through the Warburg effect, especially considering that bone is not a normoxic tissue, but a hypoxic one (5% pO2) [74], which stimulates cancer cells to switch towards a glycolytic metabolism, and therefore produce lactate and protons. Acidity is therefore an important (and often overlooked) result of the vicious cycle especially on the standpoint of bone pain.

Although the role of acidity in nociception seems clear in fields such as gastroenterology, the molecular mechanisms underlying acidity-induced bone pain are not as clear to date. Recently, studies on mouse and rat models of cancer-induced bone pain (CIBP) revealed that there are two main families of receptors that are activated by acidity: transient receptor potential channel-vanilloid (TRPVs) subfamily members and acid-sensing ion channels (ASICs) [75,76,77,78,79,80]. TRPVs and ASICs are described as proton-activated, cationic current-generating receptors, and are present in periosteal as well as bone marrow nociceptive terminals. The main molecular players belonging to this family seem to be TPRV1 and ASIC3. Although similar in function, they have different features as far as pH-sensitivity and sensitisation/desensitisation go.

TRPV1 is expressed by calcitonin gene related peptide-positive (CGRP+) C-type neural sensory terminals. It is activated by pH < 6, and sensitised to other stimuli, such as capsaicin, heat and inflammatory mediators between pH 6 and 7 [81,82]. To confirm the role of TRPV1 in CIBP, recent preclinical studies showed that treating mouse models of CIBP with either an antagonist of TRPV1 or administering another TRPV1 antagonist along with morphine, caused reduction (or further reduction in the case of co-administration) of CIBP [75,83].

At variance with TRPV1, ASIC3 is activated by milder acidosis (pH 6.7–7.3), and different models of pain showed that its activation is sufficient to induce nociceptive behaviours [84,85]. Although its role in CIBP seems clear, researchers are still to demonstrate that inhibiting its signalling can reduce CIBP in solid tumours, although a study is present where inhibiting ASIC3 is able to reduce CIBP in multiple myeloma [86]. However, an inhibitor for this channel has been developed and has been used to reduce osteoarthritic pain, and even its progression [87].

The different pH sensitivity of these two receptors convinced investigators that they are used to discriminate between mild and severe extracellular acidification of the bone marrow [88].

Recent works [86,89], along with Yoneda’s findings, showed further proof of extracellular acidification as being a driving force in CIBP. In particular, inhibiting the vacuolar proton pump (V-ATPase), used by osteoclasts to acidify the resorption lacuna by means of proton pump inhibitors (PPIs), strongly reduced CIBP in breast cancer and multiple myeloma. This is interesting from a translational point of view, since these drugs are cheap, have little side effects, and are routinely used in clinical practise for reducing stomach acidification.

### 3.2. Neurotrophins

Neurotrophins include a small family of molecules: nerve growth factor (NGF), brain-derived neurotrophic factor (BDNF) and neurotrophin-3 (NT-3). Neurotrophins have a coreceptor—p75—that is common to all of them, and a specific receptor that is tropomyosin-related kinase (Trk)-A for NGF, TrkB for BDNF and TrkC for NT-3 [90]. These are present in both the CNS and the CGRP+ sensory terminals in the bone marrow. Usually, NGF is considered as the most potent pain inducer and blocking its binding to TrkA using a monoclonal antibody (mAb911, Rinat/Pfizer) is able to strongly reduce CIBP in mouse models [91,92,93]. Tanezumab, a humanised version of mAb911, is currently in a Phase 3 clinical trial in association with opioids in a randomised, double-blind, placebo-controlled, multicenter, parallel-group study (Clinical trial NCT02609828) and is probably the drug that currently holds the most promise for pain management in bone metastatic patients, in addition to anti-resorptive drugs (read below).

BDNF could also have a role in sensitising central neurons to pain response [94], therefore general Trk inhibitors are also being developed [95]. Intriguingly, metastatic breast and prostate cancer cells express high levels of NGF and BDNF [96,97], which not only can stimulate nociceptors directly, but may also induce macrophages to secrete TNF-α, IL-6, IL-1β and prostaglandin (PG)E2 [98]. These inflammatory mediators can further activate pain responses, fuel the vicious cycle, sensitise nociceptors and activate other molecular mediators of pain, such as acidity [99,100,101]. A further link between NGF and acidity is that this neurotrophin can sensitise TRPV1 to protons and increase its expression, causing acidity-directed allodynia and hyperalgesia, which further worsens CIBP [81].

### 3.3. Inflammatory Cytokines and Chemokines

As mentioned above, ILs can increase osteoclastogenesis and tumour survival, but also induce bone pain. As a matter of fact, IL-1β, arising from cancer-related inflammation, increases macrophage expression of cyclooxygenase (COX)-2, eventually leading to an increased production of prostaglandins, which bind prostanoid receptors on sensory terminals, resulting in CIBP [102,103]. Supporting the conclusion that IL-1β is involved in CIBP, an in vivo mouse model of osteosarcoma showed that this interleukin is not only increased in the tumour area, but also in the spinal cord, and inhibiting its receptor reduces mechanical and thermal hyperalgesia [104].

TNF-α is another notable hyperalgesia-inducing substance: it has been suggested that this happens through the sensitisation of TRPV1 channels, linking back to acidity [105]. Monocyte chemoattractant protein (MCP)-1 has also been directly implicated in CIBP and mechanical allodynia and, as demonstrated by Hu and colleagues, intrathecal administration of an MCP-1 neutralising antibody reduces CIBP in a breast cancer model of bone metastasis [106]. Although other cytokines and chemokines have been correlated to bone pain [61] the link with CIBP is most likely indirect and is dependent on osteoclastic bone resorption.

### 3.4. Other Microenvironment- and Tumour-Derived Factors

Although the aforementioned factors would be sufficient to explain most of CIBP (if every hypothesis proves correct), there is still a sizeable side of it that still needs to be explained. As a matter of fact, many other molecular mediators emerged in the last few years.

One of the most important examples is arguably ATP. This molecule is present in every single living cell, and it is never found in the extracellular environment, unless there is cell or nerve damage, which may culminate in necrosis. Extracellular ATP can be sensed by the purinergic receptors P2X (ionotropic) and P2Y (metabotropic) [107]. A member of the former family, P2X3, has been studied extensively in CIBP, since it is expressed quite specifically in small diameter nociceptive fibres throughout the bone marrow and periosteum [108]. Interestingly, treating rats with antagonists for this receptor reduces pain-related behaviours in CIBP models, and the same has been shown in mouse models [108,109]. Nowadays, second generation P2X3 receptor antagonists are being developed to overcome some of the limitations encountered by the first-generation antagonists, such as dysgeusia (i.e., alteration of taste) and hypogeusia (i.e., reduction of taste) (Clinical trial NCT03449134). Nevertheless, the P2X3 antagonist Gefapixant has gone through several Phase 2 clinical trials with promising results for chronic endometriosis-related pain (ongoing, NCT03654326) and bladder pain syndrome (completed, NCT01569438), but still not CIBP.

TGF-β1 and IGF-1 are also two strong candidates for the induction of CIBP. They are highly represented in the organic bone matrix [110] and are therefore released in the microenvironment during tumour-induced bone resorption. Intriguingly, they are already notorious vicious cycle-fuelling factors [111]. A recent report showed that TGF-β1 signalling is crucial for CIBP onset and development in a preclinical study on rats [112]. This makes TGF-β1 an even more interesting molecular target for cancer bone metastases, although the ubiquitous nature of this protein makes inhibition a difficult path to walk. Nevertheless, a number of clinical trials have been completed or are ongoing, and many of them show promising results. As for IGF1, it has been shown to sensitise the TRPV1 receptor, promoting acidity-induced nociception in the context of bone metastases [113]. Another important mediator of cancer induced bone pain is osteocyte-derived Sclerostin (SOST) [114]. Osteocytes, in addition to mediating pain in response to microfractures, which are often cancer-related [76], release SOST as a consequence of multiple myeloma (MM). SOST is a potent inhibitor of the Wnt/β-Catenin pathway, which in this case appears to be a driver of MM-induced bone disease. In fact, genetically ablating SOST or blocking it with an antibody can strongly reduce osteolysis and consequent acidity-mediated pain [114].

Finally, serotonin which seems to be able to increase sensitivity of ASIC3 in the dorsal root ganglion (DRG) of rats intratibially inoculated with cancer cells [115] and to modulate TRPV1 activity as well [116].

## 4. In Vivo Models of Bone Pain

While it is relatively easy to assess pain in humans, since we can answer questions about how, how much and where we are hurting, the same cannot be said about preclinical models, such as mice and rats. These models are practical, usually have good linearity with humans, but are quite difficult to assess objectively, because of the lack of molecular markers of pain. However, over the years, investigators developed several tests of hyperalgesia and allodynia, which are easily performed on mice and rats, and can give reliable indications when done properly.

Most preclinical studies on CIBP start with a monolateral intratibial injection of cancer cells, may it be breast, prostate or multiple myeloma. DRGs are equal and symmetrical, and project to the ipsilateral limb. Therefore, performing a monolateral injection provides the perfect internal control, because it is possible to compare the ipsilateral DRG (which contains the cell bodies of the nociceptive neurons) with the contralateral DRG in the same animal. It is possible to compare quantity of proteins or transcripts such as ASICs, TRPVs, CGRP and all the other molecular markers discussed above, as well as phosphorylations, which may influence the functionality of channels such as TRPV1 [117].

Glial hypertrophy and activation is also a widely used molecular marker of pain. The most common marker used for staining activated glia is glial fibrillary acidic protein (GFAP). The staining can be performed on DRG and/or the appropriate section of the spinal cord (usually L4-L5), and glial hypertrophy can be quantified in the ipsilateral vs. contralateral DRG or posterior horn, respectively [118]. Activation of the mitogen activated protein kinase (MAPK) pathway is also commonly observed in the spinal glia of animals that feel pain. Phospho-p38 can be used as immunohistochemical marker and colocalised with a microglial marker such as CD11b to evaluate % of microglial cells with an activation of the MAPK pathway [119]. Another common way of checking for activation of pain pathways is staining for c-fos-positive second-order neurons in the posterior horn of the spinal cord, laminae I and II [77]. In CIBP models there will usually be c-fos positive neurons (meaning they are actively signalling) in the ipsilateral horn, and no positive neurons in the contralateral one. These are the most commonly used ways to check for *post mortem* evaluation of hind limb pain. Although these methods are widely used, it is usually necessary to apply a stimulus to both the inoculated and sham limb at a specific time before sacrifice, to maximise first and second order neurons activation, which might prove a source of experimental variability.

**Figure 3:**
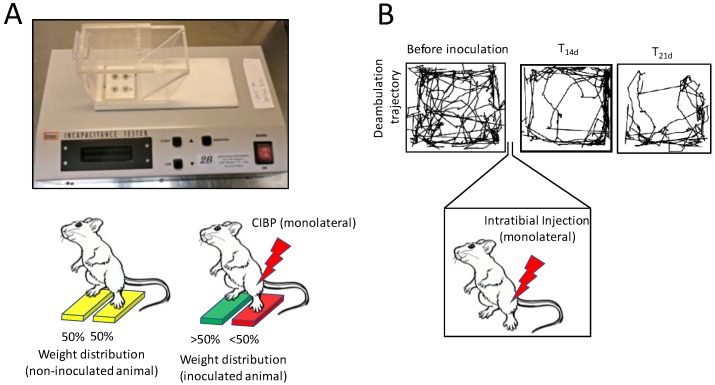
In vivo, noninvasive methods to evaluate cancer-induced bone pain (CIBP). (**A**) Incapacitance tester: cancer cells are intratibially injected monolaterally, and after an experiment-specific timeframe the animal is placed on an incapacitance tester. This is a device which features 2 scales, that are able to discriminate weight distribution between the 2 hindlimbs, when the animal is stood up at an incline (as visible from A, upper panel). In normal conditions, rodents will tend to distribute the weight evenly between the 2 limbs, but when one of them experiences CIBP, mice will relieve them from some of their body weight, reducing the % of weight bore by that limb. (**B**) Spontaneous deambulation test: mice are acclimated in a 45 × 45 × 45 cm arena 3 times the week before the cancer cells inoculation to establish a baseline. On the third test, the trajectory of the mouse is recorded and quantified over a specific timeframe (e.g., 10 min), to assess the distance the mouse is willing to walk voluntarily, without external stimulation. Cancer cells are then injected monolaterally (e.g., intratibially) and after an appropriate time (e.g., T_14_ and T_21_ days), mice will start showing a decrease in spontaneous ambulation, which is mostly due to CIBP. It is also possible to review the mouse behaviour to assess rearing behaviour and limb usage if a video recording device is used for the test.

To complement these useful but sometimes not very reliable molecular methods, a number of in vivo behavioural and performance tests to assess allodynia and hyperalgesia during its onset have been developed. One of these is the incapacitance tester [120]. This is composed by two scales, that are capable of measuring differential weight bearing between inoculated and non-inoculated limb in mice and rats (Figure 3A). The more pain the mouse is going through, the less weight it will put on the inoculated limb. This test proved to be solid, reliable and easy to perform, since mice were quite compliant. On an ethical standpoint, this is also a good option, since there is no further noxious stimulus being applied to the animal.

Another option could be the “spontaneous deambulation test” (Figure 3B; there is currently no consensus on the nomenclature of the test), which is essentially an “open field test” [121,122,123]. This consists in placing the mouse in a 45 × 45 × 45 cm (or similar) box for a fixed amount of time (usually 10 min) and measuring via software the distance the mouse walked spontaneously during the timeframe: the lower the distance, the stronger the pain. This can either be evaluated with a system of photocells or by shooting a video recording of the test, and later analysing semi-automatically via software. The latter option is preferable because it provides investigators with the chance of checking for spontaneous pain-related phenotypes as well. These include reduced limb usage, guarding and biting towards the paw of the inoculated limb. It is not surprising that this inexpensive, ethical and multiparametric analysis is becoming very popular among pain researchers [121,122,123].

Several stimulus-dependent methods are also present in literature. Among these, the von Frey test for allodynia is undoubtedly the most common one [124], also considering it is used in humans as well. This consists in stimulating the inoculated paw with a filament (or filaments) that applies an increasing amount of pressure, until the mouse withdraws the paw exhibiting a pain-related behaviour: the higher the pressure, the lower the allodynia, and vice versa. Being the most common allodynia test, very good statistical methods have been developed for data analysis, and this makes the von Frey test a good and quite reliable option when studying allodynia.

The Randall–Selitto test is also common [125]. This is performed by restraining the mouse or rat and applying increasing force to the inoculated paw using a pointy clamp-like probe. While quite similar to the von Frey method, this is usually considered to measure hyperalgesia, since the outcome measures are vocalization or paw withdrawal.

Another test that can be used in this kind of experiments is the Hargreaves test [126]. This measures heat-induced pain response in rodents. The mouse is first placed on a glass surface, and then a heat stimulus is applied to the paw through the glass (usually infrared light). The time to withdraw the paw is then recorded and longitudinal comparisons between inoculated and non-inoculated limb can give good indications about allodynia and hyperalgesia caused by CIBP [127]. A possible drawback of this test would be that hypoalgesic models can suffer tissue damage before responding to the stimulus. It is therefore important to set a maximum time for the test, and make sure that naïve mice have ~10–15 s of withdrawal time, so that there is a good time window to remove the stimulus when maximum time is exceeded.

A new generation of computer-assisted tests are recently emerging, and they hold great promise due to the possibility of analysing multiple parameters at once. The fact that no stimulus is necessary to perform the test and that they require no operator-based analysis allows to bring more objectivity into the behavioural analysis field. In the Gait analysis (Catwalk XT, Noldus) test, rodents can walk freely on an elevated glass floor, similarly to what happens with the spontaneous deambulation test. A camera placed below the floor, thanks to a sophisticated illumination system, can record the following parameters; number of paw prints, print area, paw intensity and time spent on or off a specific paw [128]. This happens automatically without any operator-based interpretation. Another interesting “next generation” pain assessment instrument is called behavioural spectrometer (Behavioural Instruments). This is a self-contained box featuring an array of photobeams and an overhead camera. Thanks to software algorithms that have been validated in both mouse and rats, the instrument is able to analyse horizontal activity, rearing activity, spontaneous paw withdrawals and other parameters with minimal human intervention [129].

## 5. Current Treatments for CIBP

Although metastatic bone disease could be asymptomatic in some cases, it has been calculated that at least 75% of cancer patients carrying bone metastases present with bone pain [130]. Several options are available to contrast CIBP; however, there is still the need to improve this treatment also because, fortunately, bone metastases have a more favourable prognosis compared to lung, liver or brain metastases, on an overall survival standpoint [131]. However, as they say, “surviving is not the same as living”, and very often bone metastases are extremely painful, and significantly degrade the quality of life of oncologic patients [132]. In the next paragraphs the current options for treating CIBP are reported.

### 5.1. Surgical Intervention

This procedure has a double advantage, since it allows patients to regain motor activity as well as to relieve pain. Surgical intervention is strongly indicated in case of vertebral metastases, where tumour mass or fractures induce vertebral compression. According to the severity of the disease, different options are implemented. When prognosis is poor, for example, usually a palliative spinal decompression followed by radiation therapy is advisable, while for patients with longer survival probability more drastic surgery may be undertaken, to achieve longer-term benefits [133]. Surgical intervention is usually performed along with a palliative treatment with corticosteroids. Less complex intervention is performed in case of pathological fractures, by applying an internal fixation followed by radiation therapy [133]. However, when patients are highly debilitated, an external fixation may be performed, which could be able to counteract bone pain [134].

Among surgical procedures, neurodestruction should be also mentioned [135]. This is mainly used for patients resistant to palliative pharmaceutical treatment and can be performed in different districts. Spinal cordotomy severs the spinothalamic tract, which results in complete lack of pain signalling from the innervated portion. However, since this procedure results in complete loss of pain sensation, there could be complications such as ulcerations or accidental burns because the patient does not perceive the stimulus as noxious [135,136]. Two other options are thalamotomy and cingulotomy, the former disrupting the somatosensory area of the thalamus and the latter involving the limbic system. These are all very invasive options that should be used only when treatments with major opiates (e.g., morphine) have proven inadequate.

### 5.2. Radiotherapy

Radiotherapy is an option that may prove efficacious for both tumour mass reduction and pain relief. In case of bone metastases, this therapy can promote ossification of osteolytic lesions, which stabilizes the affected bone [137]. A desensitisation of nerve terminals has also been documented, and usually treated patients show pain relief within two weeks from the start of treatment [137,138]. Three radiotherapy options can be usually applied: external-beam radiation therapy (EBRT), stereotactic body irradiation therapy (SBRT) and radionuclide treatment [135]. As reported in McQuay et al., and Ahmad et al., EBRT induces a complete pain relief in 24% patients and 50% relief in 41% patients [135,139]. Similar results were reported by Nomiya and colleagues [140].

Radioisotopes are particularly indicated for osteosclerotic bone metastases and they include ^89^Strontium (Sr), ^153^Samarium (Sm) and ^223^Radium (Ra) [141,142]. The latter proved to be very efficacious, likely due to its high affinity for the bone, thus minimizing side effects in other organs. This radionuclide emits short-range high-energy α-particles, which are very low-penetrating, thus causing strong, but very well-localised cell damage. Moreover, in Phase II clinical studies it proved to significantly improve overall survival as well as pain relief [143].

### 5.3. Drug Treatment

Treatment options change according to the intensity of the pain, since usually for mild pain an anti-inflammatory therapy is applied, while for severe pain opioid treatment is necessary, according to the WHO 3-step pain ladder.

#### 5.3.1. Nonsteroidal Anti-Inflammatory Drugs

These include COX-1/COX-2 inhibitors which, as above described, block the production of PGEs and consequently their binding to prostanoid receptors on sensory terminals, resulting in inhibition of CIBP. COX-2 is also highly expressed by cancer cells, and therefore these inhibitors could also have an anti-tumoural activity besides the effect on CIBP [144,145]. However, it seems that COX-2 specific inhibitors increase susceptibility to myocardial infarction, while not being more effective than general COX inhibitors [146]. These clinical results show that more careful studies are needed to clarify the mechanism of action and reduce adverse effects.

#### 5.3.2. Opioid Treatment

This option is also widely employed in CIBD especially when bone pain is severe. Indeed, at least 80% of cancer patients undergo this treatment [132]. Opioids employed in CIBD mainly act through the interaction with µ-receptors [147]. Side effects of this treatment are nausea, constipation, addiction, excitement and, last but not least opioid receptor desensitisation, which occurs after prolonged use.

#### 5.3.3. Anti-Resorptive Agents

Bisphosphonates and denosumab are drugs successfully employed in the clinical practice to treat osteoporosis, by inducing osteoclast apoptosis or inhibiting osteoclast formation, respectively [148]. They have also been introduced as an adjuvant therapy to reduce the frequency and delay the appearance of SREs [149,150]. However, their specific role in analgesia to relief bone pain is still debated. From one side, some phase II studies showed a positive effect [151,152], however side effects, including osteonecrosis of the jaw, neutropenic fever and pyrexia are known [153,154]. Indeed, both pamidronate and zoledronate reduced skeletal morbidity [47], and approximately 50% of patients reported an improvement of bone pain relief [155], while Denosumab presented with a higher efficiency compared to bisphosphonates in preventing SREs [46,156].

Since the primary effect of these drugs is to impair osteoclastic bone resorption, these treatments indirectly inhibit acidification of the bone microenvironment, thus reducing the activation of acid-sensing channel and subsequently bone pain. Finally, a recent meta-analysis performed by Posta-Sales and colleagues concludes that although the analgesic effect of bisphosphonates and denosumab seems to be weak, it can instead be useful to prevent pain by delaying its onset [157]. This is particularly true for cancer patients with a long life expectancy, since in this case the treatment could postpone bone pain.

Based on this data, for these bone-targeted compounds it is recommended to start the treatment as soon as possible once the bone metastasis has been identified, even if it is still asymptomatic [133].

#### 5.3.4. Endothelin-A (ET-A) Receptor Antagonists: a New Avenue

ET-A receptor antagonists are a class of compounds that show great promise in pain treatment, particularly for prostate cancer-induced bone metastases, which is conceivable if we consider the pivotal role played by the ET-1 pathway in this contest. Besides its protumoural and proangiogenic effect, ET-A also stimulates hyperalgesia, through sensitisation of primary afferent nociceptors. This happens through different mechanisms, which are thought to include calcium as second messenger in the primary nociceptor, which eventually results in sensitisation of TRPV1 and other pain-mediating channels and pathways, such as the β-endorphins–μ-opioid receptor (MOR) pathway [158]. Consistently, ET-A receptor antagonists show an analgesic effect [159] and also induce the activation of the opioid signalling by promoting the release of β -endorphins [160]. This treatment is a very attractive option as a “next-generation” drug because it can tackle both tumour growth and CIBP at the same time. Although clinical trials are still inconclusive, we hope this, and other targeted therapies will become available in the future to provide oncologists with numerous superior options.
